# Reversible flowering of CuO nanoclusters via conversion reaction for dual-ion Li metal batteries

**DOI:** 10.1186/s40580-022-00353-3

**Published:** 2023-01-13

**Authors:** Siying Li, Jung-Hun Lee, Soo Min Hwang, Young-Jun Kim

**Affiliations:** 1 School of Mechanical and Automotive Engineering, Guangxi University of Science and Technology, Liuzhou, 545616 China; 2 SKKU Advanced Institute of Nanotechnology (SAINT), Sungkyunkwan University, Suwon, 16419 Republic of Korea; 3 SKKU Institute of Energy Science and Technology (SIEST), Sungkyunkwan University, Suwon, 16419 Republic of Korea

**Keywords:** Non-flammable, SO_2_-in-salts electrolyte, Cupric oxide, Nano-structuring, Polyacrylonitrile, Conversion reaction

## Abstract

Dual-ion Li metal batteries based on non-flammable SO_2_-in-salt inorganic electrolytes ( Li–SO_2_ batteries) offer high safety and energy density. The use of cupric oxide (CuO) as a self-activating cathode material achieves a high specific capacity with cost-effective manufacturing in Li–SO_2_ batteries, but its cycle retention performance deteriorates owing to the significant morphological changes of the cathode active materials. Herein, we report the catalytic effect of carbonaceous materials used in the cathode material of Li–SO_2_ batteries, which act as templates to help recrystallize the active materials in the activation and conversion reactions. We found that the combination of oxidative-cyclized polyacrylonitrile (PAN) with N-doped carbonaceous materials and multi-yolk-shell CuO (MYS-CuO) nanoclusters as cathode active materials can significantly increase the specific capacity to 315.9 mAh g^− 1^ (93.8% of the theoretical value) at 0.2 C, which corresponds to an energy density of 1295 Wh kg_CuO_^−1^, with a capacity retention of 84.46% at the 200th cycle, and the cathode exhibited an atypical blossom-like morphological change.

**Supplementary Information:**

The online version contains supplementary material available at 10.1186/s40580-022-00353-3.

## Introduction

Recently, the progress in Li-ion batteries (LIBs) has led to the development of new devices such as electric vehicles, mobile communication devices, and energy storage systems. These devices require high energy densities and long lifespans of LIBs. However, LIBs with organic electrolytes still present safety concerns as they can catch fire or explosion because of the high flammability of their electrolytes. Moreover, next-generation high-energy-density rechargeable batteries such as Li–S and Li-air batteries present similar safety issues because they use flammable organic electrolytes [1, 2].

In 2015, Jeong et al. revisited inorganic SO_2_-in-salt electrolytes and promoted the development of next-generation Li or Na secondary batteries with SO_2_-in-salt electrolytes [3]. The ionic conductivity and flammability of SO_2_-in-salt electrolytes comprising lithium or sodium tetrachloroaluminate (LiAlCl_4_ or NaAlCl_4_, respectively) dissolved in liquid SO_2_ are higher and lower, respectively than those of conventional organic electrolytes [4–6]. Copper(II) chloride (CuCl_2_) has been used as a cathode material for SO_2_-in-salt battery systems because it is inexpensive and its high specific capacity is comparable to that of the current LIB cathode materials [7, 8]. In addition, the voltage difference between the discharge and charge curves (Δ*V*) for the CuCl_2_-to-CuCl phase transition in SO_2_-in-salt secondary battery systems is negligible (approximately 0.11 V at 0.25 C) compared with those of other battery systems [7]. Although the energy density of CuCl_2_ cathodes (~ 580 Wh kg^− 1^) and voltage efficiency during charging-discharging are high, their commercialization is hindered by their high moisture reactivity. To address this issue, Kwak et al., Kim et al., and Li et al. used copper metal and copper oxides as cathode materials, which were converted to CuCl or CuCl_2_ through a self-activation process before the initial charging or discharging began [9–11]. However, the self-activation process leads to a large volume expansion of the cathode (e.g., conversion of CuO to CuCl_2_ is accompanied by 224% volume expansion), which is responsible for the low initial capacity and degraded cycle retention of copper metal or copper oxide cathodes [10, 11].

To date, numerous researchers have focused on nanoengineering conversion-type active materials for rechargeable batteries [12–14] Nanoengineering methods can be classified into four categories: downsizing, forming hollow or hierarchical structures, and hybridization with nanoscale carbonaceous materials [13]. Nanosized active materials exhibit excellent electrochemical performance owing to their large surface areas and short Li^+^ ion diffusion lengths, facilitating fast charging [15–18]. Furthermore, the hollow structure can effectively alleviate the stress caused by volume changes by providing adequate space [19–22]. The structures developed by combining the advantages of the two aforementioned nanoengineering methods present a hierarchical configuration. In addition, such structures were hybridized with carbonaceous materials to provide smooth electron transfer pathways [23, 24]. Moreover, the crystallographic orientation of the active materials can be tuned using the carbon coating utilized as the template [25]. These methods can be used to develop Cu-based self-activating cathodes in SO_2_-in-salt secondary batteries. Kwak et al. evaluated the performance of copper(I) oxide (Cu_2_O) with several nanostructures including mesoporous nanospheres, nanocubes, and nano-octahedrons [9]. Copper(II) oxide (CuO) with nano- and microstructures as cathode materials in SO_2_-in-salt secondary battery systems has been studied as well [11]. They reported that nanostructuring improved the specific capacity but did not affect the cycling performance of the cathodes because the active cathode material underwent significant micro-shape changes in addition to volume changes during cycling. Therefore, new methods are required to develop Cu-based cathodes with high energy densities and cycling stabilities for SO_2_-in-salt secondary batteries.

Herein, we developed a hybrid nanoengineering-based method to fabricate high-performance cathodes for SO_2_-in-salt secondary battery systems. As the cathode material, multi-yolk-shell CuO (MYS-CuO), with a microsphere structure wrapped with nanoparticle clusters, was coated with cyclized polyacrylonitrile (PAN) and 0-D ketjen black (KB) as the conducting agent. The self-activation and charge-discharge reactions of the active materials were influenced by their initial morphology and the coated cyclized PAN and conducting KB, leading to interesting morphological changes and enhanced electrochemical properties. The reversible specific capacity of the cathode reached a high value of 315.9 mAh g^− 1^ (93.8% of the theoretical value) at 0.2 C (1 C = 300 mAh g^− 1^ for CuO) and remarkable capacity retention of 84.46% at the 200th cycle. We believe that our results will promote the commercialization of Li metal dual-ion batteries with non-flammable SO_2_-in-salt inorganic electrolytes.

## Methods/experimental

### Materials

Copper (II) acetate monohydrate (Cu(CHCOO)_2_·H_2_O, 98.0–102.0%), sodium hydroxide (NaOH, 98%) from Alfa Aesar, and L-ascorbic acid from Sigma-Aldrich were used for the fabrication (MYS-CuO). Lithium chloride (LiCl, 99.9%, ultra-dry), aluminum chloride (AlCl_3_, 99.99%, ultra-dry), from Alfa Aesar, and sulfur dioxide (SO_2_, 99.5%) purchased from Alpha Gas were used for LiAlCl_4_·3SO_2_ preparation. Poly(tetrafluoroethylene) (PTFE, 60 wt% emulsion in H_2_O) and polyacrylonitrile (PAN, Mw 150,000) from Sigma-Aldrich, 1-Methyl-2-pyrrolidone (NMP, 99.5%) from Samchun Chemicals, and Ketjen Black (KB, EC-600JD carbon black) from Akzo Nobel, Japan, were used for cathode fabrication. Glass microfiber (GF, 190 μm thickness, GC50) obtained from Advantec was used as the separator. Thionyl chloride (SOCl_2_, > 99.5%) was purchased from Daejung Chemicals to wash out the LiAlCl_4_·3SO_2_ electrolyte remaining on the electrode after disassembling the cell.

### Preparation of multi-yolk-shell CuO microspheres

Multi-yolk-shell CuO microspheres (MYS-CuO) were used as the active materials. Mesoporous Cu_2_O microspheres were then fabricated. NaOH pellets were dissolved in 165.6 mL deionized water to make the solution pH 11.3. The solution turned light blue when 50.4 mL of 0.3 mol L^-1^ Cu(CH_3_COO)_2_ aqueous solution was added to the solution under stirring at 400 rpm. With the dropwise addition of 54.0 mL of 0.28 mol L^-1^ ascorbic acid (AA) aqueous solution, the solution first changed to a brown turbid solution, and the suspension became yellow and saffron, revealing the formation of Cu_2_O mesoporous microspheres. The mixture was stirred at room temperature for 20 min, collected, and washed with deionized water and ethanol via vacuum filtration. The saffron powders were dried overnight in an 80 °C vacuum oven, followed by calcination at 360 °C for 10 h in a box furnace to obtain MYS-CuO. During the heat treatment of mesoporous Cu_2_O in air, the outward diffusion of copper atoms was faster than the inward diffusion of oxygen atoms, resulting in the hollow structure of CuO as a calcination product.

### Preparation of SO_2_-in-salt electrolyte

AlCl_3_ was stored in an Ar-filled ampule without further purification. The LiCl was dried in a vacuum oven at 120 °C for 24 h before use. AlCl_3_ and LiCl were mixed at a molar ratio of 1:1.1 and sealed in a glass/Teflon vessel in an Ar gas-filled glove box (< 1 ppm H_2_O and O_2_). An additional 10 mol% of LiCl was added to mitigate the formation of free AlCl_3_, which is corrosive to metals. SO_2_ gas was blown into the vessel at a pressure of 1.5 bar. Subsequently, the white powder mixture became a transparent light-ochre-colored liquid. The SO_2_ concentration was determined by weighing the vessel before and after SO_2_ blowing. Finally, the fabricated electrolyte was transferred to a pre-vacuum-dried glass vial (120 °C, 24 h) in a humidity-controlled Ar gas-filled glove box (< 1 ppm H_2_O and O_2_). A small piece of Li metal was added to the electrolyte to remove the AlCl_3_ and water residues. Considering SO_2_ as the solvent, the molarity of LiAlCl_4_ was 5.2 M.

### CuO cathode fabrication with PAN

The as-prepared MYS-CuO (active material) and KB (conducting material) were first mixed at a weight ratio of 6:2 in a mortar with a pestle. The PAN/NMP solution was then added to the mixture and homogenized using a planetary mixer (AR-100, Thinky) at 2000 rpm. The weight ratio of MYS-CuO, KB, and PAN was controlled at 6:2:1. The slurry was cast onto a clean glass plate with a 5 cm doctor blade and annealed in ambient air at 200, 250, and 280 °C for 1 h at a heating rate of 2 °C min^-1^ to cyclize the PAN after drying overnight in a vacuum oven at 120 °C. The powder was collected and mixed with PTFE (60 wt% emulsion) using a mortar and pestle to form a clay-like paste. The final composition of the CuO: KB: PAN: PTFE was 6:2:1:1. The thickness of the clay-like paste was controlled to approximately 100 μm using a press-gap-adjustable roller press, and punched into Φ6 discs. The loading mass of the electrode was 10–11 mg cm^-2^ with a density of approximately 1 g cc^-1^. The electrodes were dried in a vacuum oven at 120 °C for more than 12 h prior to coin cell fabrication.

### Electrochemical measurements

A CR2032 coin-type cell was assembled in a dry room (dew point < − 58 °C) using CuO as the working electrode, a 150 μm-thick Li metal sheet as the counter electrode, 200 µL LiAlCl_4_·3SO_2_ as the electrolyte, and a 190 μm-thick glass microfiber membrane as the separator. Electrochemical tests were performed using Neware (BTS-4008-5V10mA) and Toyo (TOSCAT-3100) battery testing systems at room temperature (25 °C). The cells were operated galvanostatically at 62.5 mA g_CuO_^-1^ within the voltage range of 3.2–3.65 V vs. Li/Li^+^. In this study, the capacity and voltage were calculated based on the mass of the CuO cathode material. For the rate capability test, the charge and discharge rates were varied from 0.1 to 1 C (1 C = 300 mA g^-1^). Electrochemical impedance spectroscopy (EIS) measurements were performed using a biologic VSP-100 in the frequency range of 1 MHz to 10 mHz with a voltage perturbation of 5 mV. The electrodes were immersed in the electrolyte for 1 week to activate CuO to CuCl_2_, and then assembled symmetrically in coin cells for EIS measurement.

### Characterization

FE-SEM with energy-dispersive X-ray analysis (S-4700, HITACHI) and high-resolution TEM (JEM-2100 F, JEOL) were used to examine the morphology, microstructure, and composition of the as-prepared CuO and its electrodes. For ex situ FESEM observation of CuO electrodes after resting or cycling, the electrode was carefully removed from the coin cell and rinsed with SOCl_2_ in an Ar-gas-filled glove box (< 1 ppm H_2_O and O_2_) to remove the electrolyte residue. For the ex-situ XRD (D8 DISCOVER, Bruker) measurements, the CuO electrodes were vacuum packed in a polythene sealing bag to prevent phase transformation in the atmosphere because LiCl, CuCl_2_, and CuCl, as electrode resting and cycling products, are sensitive to moisture and air. The surface chemistry of the CuO electrodes after resting was examined using XPS (Thermo Fisher Scientific Co. Inc.). All the XPS spectra were shifted by the same values based on the C 1s peak at 284.8 eV.

## Results and discussion

### Limitations of CuO cathode

The separation of the active materials in SO_2_-in-salt systems with self-activating Cu-based cathodes, which can occur during the self-activation reaction and cycling, is attributed to the significant changes in their microstructure, leading to capacity fading [11]. The conversion of the inactive CuO phase into the active CuCl_2_ phase via spontaneous chlorination is thermodynamically favorable. The chlorination reaction of CuO in SO_2_-in-salt electrolytes can be expressed as follows:

1 $$3CuO+2LiAlC{l}_{4}\to 3CuC{l}_{2}+2LiCl+A{l}_{2}{O}_{3}\varDelta {G}^{0}=-342.48 kJ mo{l}^{-1}$$

where Δ*G*^0^ is the calculated Gibbs free energy change for the chlorination reaction.

As described in Additional file 1, CuO spontaneously reacts with LiAlCl_4_ in the electrolyte to form thermodynamically stable CuCl_2_, LiCl, and Al_2_O_3_. Moreover, CuO with a monoclinic distorted tetragonal PtS structure transforms into CuCl_2_ with a CdI_2_-like layered structure via an anion exchange reaction (Additional file 1: Fig. S1) [26]. The change in the crystal structure and volume expansion during the anion exchange reaction can cause unavoidable morphological changes and separation of the active materials [11].

Moreover, separation of the active materials occurs during the conversion reactions associated with the charge-discharge process. Typically, when the active materials are optimized using nanostructuring strategies, it is expected that their original morphology will be maintained even if their crystal structure changes. Most conversion-type active materials (M_a_X_b_; M = transition metal, such as Mn, Fe, Co, Ni, and Cu; X = anion, such as O, S, Se, F, N, and P) store Li^+^ ions via two-step reactions [27, 28]. During the first lithiation step, Li^+^ ions are inserted into the crystal lattice of the active material to form intermediate ternary Li–M–X phases as follows:

2 $${{M}_{a}X}_{b}+(b\cdot n)Li\leftrightarrow L{i}_{b\cdot n}{{M}_{a}X}_{b},$$

where *n* is the formal oxidation state of x. During the second lithiation step, as the reaction proceeds gradually, a transition metal precipitates and lithium compounds are formed, as follows:

3 $$L{i}_{b\cdot n}{{M}_{a}X}_{b}\leftrightarrow aM+ {bLi}_{n}X$$

Owing to the formation of the Li–M–X intermediate phase, the shapes of the M_a_X_b_ active material and the transition metal did not change significantly. In contrast, CuCl_2_ with a monoclinic structure reacts with Li in the SO_2_-in-salt electrolyte system to form CuCl with a zinc-blende structure and LiCl with a cubic structure, as follows: [9–11]

4 $$Li + Cu{Cl}_{2} \leftrightarrow CuCl+ LiCl$$

The ionic radii of anions are larger than those of cations; hence, they play a critical role in determining the shape of the crystal lattice. Therefore, when Cl^−^ ions deviate from the original crystal lattice and form a new phase, the remaining atoms form a new crystal structure. Consequently, the morphology of the active materials changes continuously during the redox reaction between CuCl_2_ and CuCl, which occurs during cycling and is accompanied by separation of the active materials.

### Effect and modification of carbon materials and CuO

**Fig. 1:**
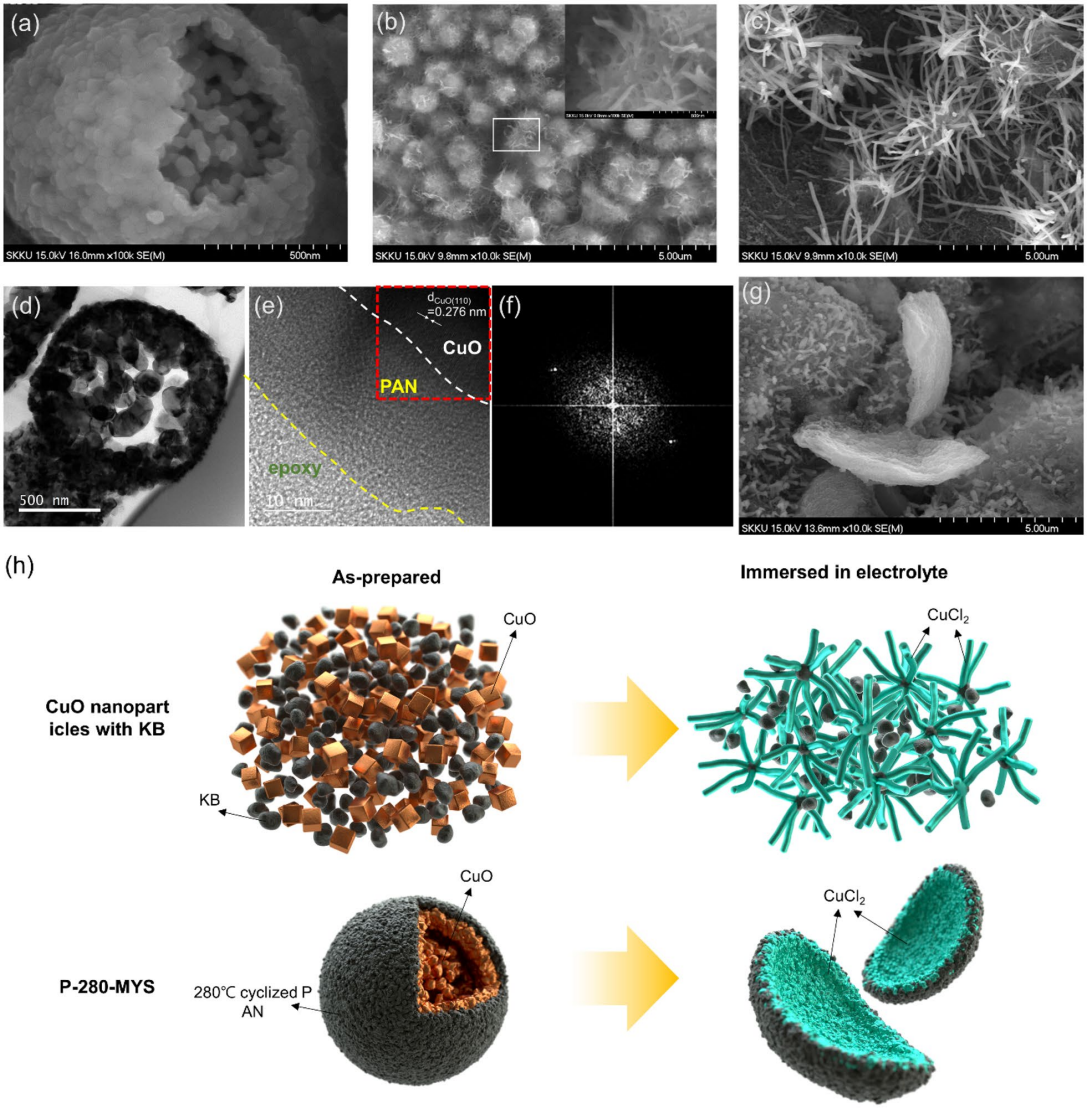
Field-emission scanning electron microscopy images of the **a** as-prepared multi-yolk–shell CuO (MYS-CuO) microspheres, **b** carbon-free MYS-CuO cathode (CuO: polytetrafluoroethylene (PTFE) = 6:2), and **c** Ketjen black (KB)-containing MYS-CuO cathode (CuO: KB: PTFE = 6:2:2) after immersion in the electrolyte. **d** Transmission electron microscopy (TEM), and **e** high-resolution TEM images of polyacrylonitrile (PAN)-coated MYS-CuO microspheres cyclized at 280 °C. **f** Fast Fourier transform pattern of the area enclosed by the red dashed square in **e**. **g** KB-and-PAN-containing MYS-CuO cathode cyclized at 280 °C (CuO: KB: PAN: PTFE = 6:2:1:1) after immersion in the electrolyte. **h** Schematic of the structural transformation of the active cathode materials: without carbon coating (top row), with carbon coating (bottom row), before immersion in the electrolyte (left column), and after immersion in the electrolyte (right column). The black and tan squares represent KB particles and MYS-CuO microspheres, respectively

As indicated in Additional file 1: Fig. S2c–f, KB, the 0-D conductive additive promotes the fast conversion of the evenly distributed nanosized CuO particles into CuCl_2_. However, the resulting rapid change in shape led to the separation of the active material and block the conductive path because insulating CuCl_2_ covered the conductive KB nanoparticles. In this study, we used CuO microspheres as the active material instead of uniformly distributed CuO nanoparticles. Field-emission scanning electron microscopy (FE-SEM) image of the as-prepared MYS-CuO microspheres is presented in Fig. 1a and Additional file 1: Fig. S4. The MYS-CuO microspheres exhibited a non-porous outer shell structure at ~ 1 μm in diameter and a partially hollow internal structure comprising nanoparticle clusters (~ 50 nm in size) inside the cavities. The conversion of CuO into CuCl_2_, which was accelerated by KB, progressed inward from the outside of the MYS-CuO clusters. Therefore, the effect of conductive KB on the conversion reaction was negligible, even though KB and CuO nanoparticles were homogeneously mixed.

The carbon-based conductive agent surrounding the active material played a catalyst-like role, accelerating the self-activation reaction. The FE-SEM images of the carbon-free and several carbon-containing CuO cathodes after immersion in LiAlCl_4_·3SO_2_ are presented in Fig. 1b, c, g, and S2c–h. After immersion for 48 h, short CuCl_2_ needles grew on the surface of the spherical CuO particles of the carbon-free cathode (Fig. 1b and S2c), and the chemical composition of the needles was confirmed using X-ray diffraction (XRD) (Additional file 1: Fig. S3). Moreover, as the spherical CuO nanoparticles were consumed, wire-shaped CuCl_2_ particles were formed at the KB-containing cathode (Fig. 1c, Additional file 1: Fig. S2e, and S3). Owing to its high surface area, 0-D KB provided abundant active sites. Furthermore, KB served as the catalyst and template for the recrystallization of CuCl_2_ nanowires with low surface energy [29]. This reaction was similar to the vapor-liquid-solid growth of 1-D structures, thus facilitating the self-activation of the cathode material. However, such changes in the morphology of the active cathode material electrically isolated it from the conductive agent, leading to the deterioration of the electrochemical properties of the cathode. In addition, the large volume changes during repeated charging–discharging reduced the physical contact between the cathode components, and consequently, the cathode cracks.

To mitigate this volume change, we mixed a solution of PAN in N-methyl-2-pyrrolidone with MYS-CuO and KB to ensure robust conduction pathways. The mixture was subjected to cyclization at 280 °C under ambient air conditions. The cross-sectional microstructure of the PAN-coated MYS-CuO cyclized at 280 °C (P-280-MYS) was analyzed using focused ion beam microscopy and transmission electron microscopy, respectively, and the results are presented in Fig. 1d and e, respectively. The (100) plane of monoclinic CuO can be observed in the fast Fourier transform pattern of the region enclosed by the dashed red square in Fig. 1e that is illustrated in Fig. 1f. The FE-SEM images and XRD profile of the P-280-MYS cathode after immersion in the electrolyte (Fig. 1g and Additional file 1: Fig. S2g and Fig. S3, respectively) revealed the presence of mossy-like and sprout-shaped CuCl_2_ particles, indicating that cyclized PAN, which is a carbon material containing N-doped delocalized sp^2^ π-bonds, affected the formation of the CuCl_2_ phase via the conversion reaction at the cathode. A schematic of the structural transformation of carbon-containing CuO particles and MYS-CuO cathodes during immersion in the electrolyte is depicted in Fig. 1h.

Similar changes in morphology were observed for cathodes comprising hollow CuO nanocubes (CuO HNCs) as the active material with similar cathode compositions. (Additional file 1: Fig. S2 b, d, f, and h) However, the CuCl_2_ particles of the cyclized PAN-containing CuO HNC cathode were plate-shaped (Additional file 1: Fig. S2h) unlike the sprout-shaped CuCl_2_ particles of the P-280-MYS cathode, which was attributed to the initial shape of the CuO nanoparticles.

### Characterization of oxidative cyclized PAN

The oxidative cyclization of PAN via heat treatment at 200–300 °C under ambient air converted the polymer into pyrogenic carbon. During this process, the nitrile (C ≡ N) groups of the PAN chains were converted into C = N and C–N bonds, resulting in a cross-linking ring structure with pyridinic, pyrrolic, and quaternary N atoms. The extent of cyclization depended on the reaction temperature.

**Fig. 2:**
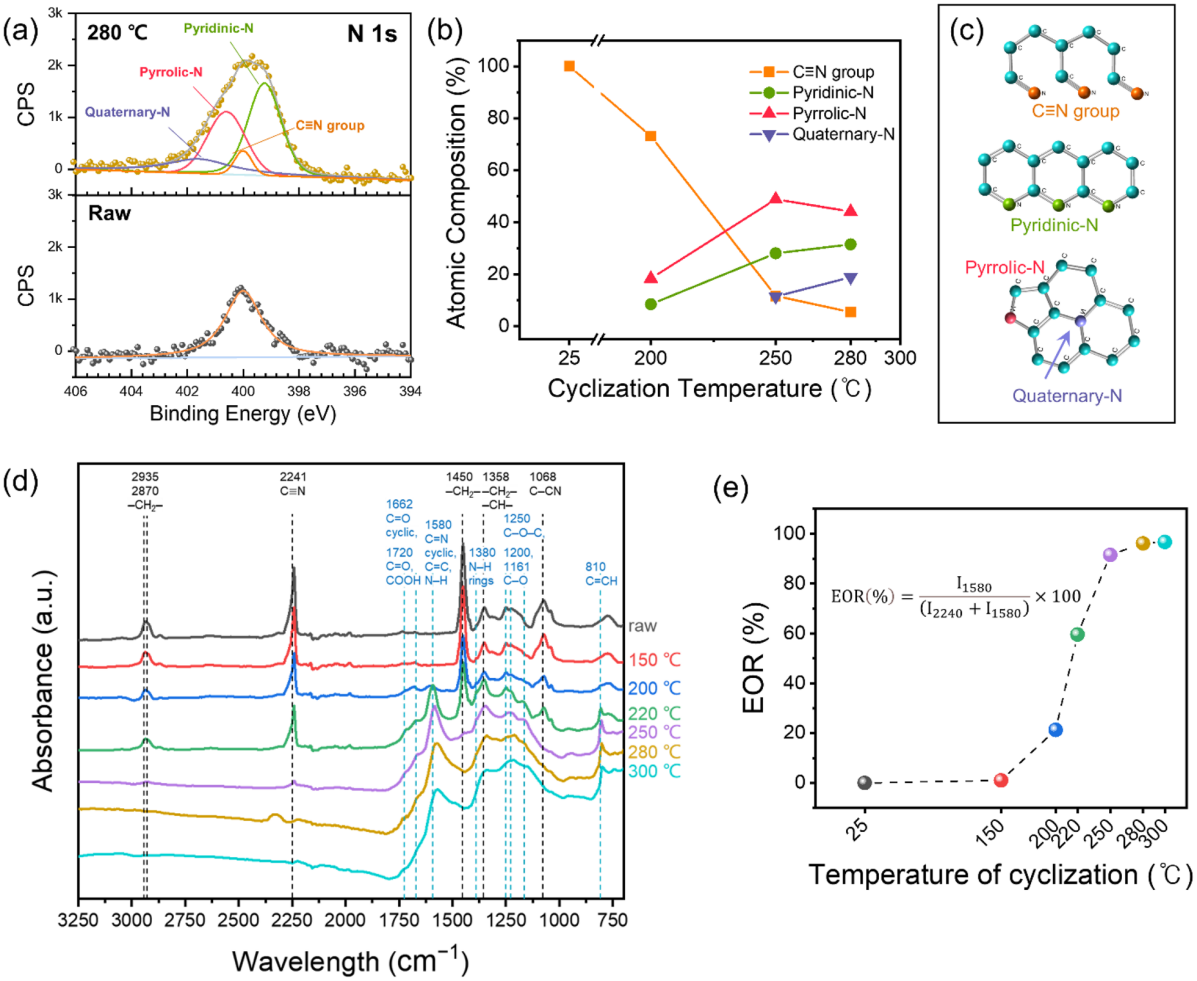
Characterization of PAN-containing CuO cathodes and PAN powder before and after cyclization at several temperatures. **a** N 1s X-ray photoelectron spectroscopy (XPS) profiles of CuO cathodes with non-cyclized PAN and 280 °C cyclized PAN, marked with “Raw” and “280 °C”, respectively. **b** Atomic composition of N-containing functional groups at different cyclization temperatures according to their N 1s XPS profiles. **c** Structures of C ≡ N and pyridinic-, pyrrolic-, and quaternary-N groups. **d** Fourier-transform infrared (FTIR) spectra of the raw and cyclized PAN powder at temperatures in the range of 150–300 °C. **e** Extent of ratio (EOR) of the PAN cyclization reaction calculated using FTIR data

To analyze the relationship between the chemical structure and cyclization temperature of the PAN-containing MYS-CuO cathodes, we performed X-ray photoelectron spectroscopy (XPS) and Fourier-transform infrared (FTIR) spectroscopy experiments. The N 1s XPS profiles of the MYS-CuO cathodes with non-cyclized PAN (P-MYS) and PAN cyclized at 280 upplementary Infor°C (P-280-MYS) are illustrated in Fig. 2a–c and Additional file 1: Fig. S5. The only peak at 400.0 eV in the N 1s XPS profile of the P-MYS cathode was attributed to the presence of C ≡ N groups. After cyclization, this peak was broadened by the evolution of three new peaks at 399.2, 400.6, and 401.6 eV, which were ascribed to pyridinic-, pyrrolic-, and quaternary-N, respectively. The intensities of these peaks increased with increasing cyclization temperature, whereas the intensity of the peak corresponding to the C ≡ N groups decreased (Fig. 2b and Additional file 1: Fig. S5). The structures of C ≡ N and pyridinic-, pyrrolic-, and quaternary-N groups are shown in Fig. 2c. The peaks in the N 1s XPS profile were upshifted by 0.5 eV compared with the values reported in the literature [30, 31]. The shift was attributed to the strong interaction between the N species and Cu(II) sites of CuO [32–34].

The FTIR spectra of the raw and cyclized PAN powders at temperatures between 150 and 300 °C are presented in Fig. 2d. As the oxidative cyclization temperature increased, the intensities of the peaks at 2241 cm^-1^ (C ≡ N group), 2935, and 2870 cm^-1^ (C–H stretching of the –CH_2_– groups of the PAN backbone), 1450 cm^-1^ (C–H bending vibrations of the –CH_2_– groups of the PAN backbone), and 1358 cm^-1^ (C–H bending vibrations of the –CH_2_– and –CH– groups of the PAN backbone) decreased. Three new bands at 1580 cm^-1^ (attributed to the stretching of the C = C bonds, cyclic C = N bonds, and in-plane bending of the N–H bonds), 1380 cm^-1^ (assigned to the bending vibration of the C–H and N–H bonds in the rings), and 810 cm^-1^ (ascribed to the C = CH bonds of the aromatic rings) emerged at 200 °C. The intensities of these bands increased with increasing cyclization temperature, revealing that the C ≡ N groups were converted into a cyclic structure when the temperature exceeded 200 °C. The extent of reaction (EOR) for the cyclization of PAN with respect to the heat treatment temperature (Fig. 2e) was calculated as follows:

5 $$EOR \left(\text{\%}\right)=\frac{{I}_{1580}}{({I}_{1580}+{I}_{2241})}\times 100$$

where ${I}_{1580}$ and ${I}_{2241}$ are the intensities of the bands at 1580 and 2241 cm^-1^, respectively. The EOR was approximately 100% at temperatures higher than 280 °C. The differential scanning calorimetry profile of PAN revealed that it underwent pyrolysis, and exothermic peaks were observed at temperatures higher than 300 °C (Additional file 1: Fig. S6).

The presence of O during the oxidative cyclization reaction led to the formation of numerous O-containing groups.(Fig. 2d) In particular, bands at 1720 cm^-1^ (ascribed to the stretching of the C = O bonds in ketones, aldehydes, and –COOH groups), 1662 cm^-1^ (attributed to the stretching of the C = O bonds in the highly conjugated acridone rings), 1161–1200 cm^-1^ (corresponding to the stretching of the C–O bonds), and 1250 cm^-1^ (attributed to the asymmetric stretching of the C–O–C bonds) were observed in the FTIR spectra of the PAN-containing MYS-CuO cathodes cyclized at temperatures higher than 200 °C.

### Characterization of PAN-coated MYS-CuO cathodes

**Fig. 3:**
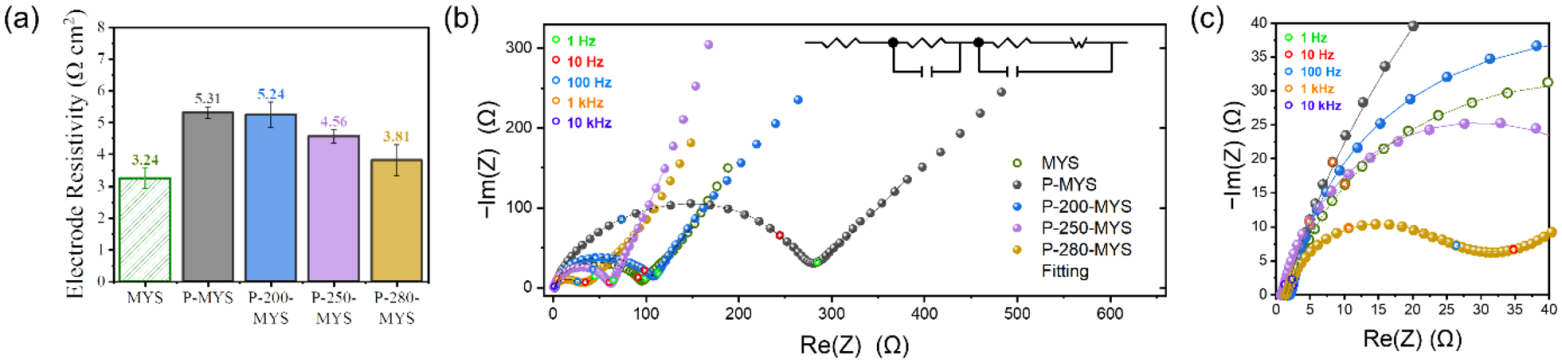
**a** Resistivity of the as-prepared multi-yolk–shell (MYS)-CuO cathodes with non-cyclized PAN (P-MYS; CuO: KB: PAN: PTFE = 6:2:1:1), with PAN cyclized at 200, 250, and 280 °C (P-200-MYS, P-250-MYS, and P-280-MYS, respectively; CuO: KB: PAN: PTFE = 6:2:1:1), and without PAN (MYS, CuO: KB: PTFE = 6:2:2). **b** Nyquist plots of the symmetric cells featuring different cathodes after resting for 1 week. **c** High-frequency region of the Nyquist plots in **b**

The N-doping of carbon materials could increase their electron transport rate, electron shuttling efficiency, and electron carrier concentration. The resistance of the P-280-MYS cathode was as low as that of the KB-containing MYS-CuO cathode ( CuO: KB: PTFE = 6:2:2). The resistivity of the cathode containing non-cyclized PAN (P-MYS, CuO: KB: PAN: PTFE = 6:2:1:1) was high (5.31 Ω cm^2^; Fig. 3a). As the cyclization temperature was increased from 200 °C (P-200-MYS) to 250 °C (P-250-MYS), and 280 °C (P-280-MYS), the cathode resistivity decreased to 5.24, 4.56, and 3.81 Ω cm^2^, respectively (Fig. 3a). These results demonstrated that the formation of N-doped carbon with delocalized sp^2^ π-bonds during PAN cyclization improved the electrical conductivity of the cathode.

To evaluate the charge transfer capability of the cathodes after resting, we performed electrochemical impedance spectroscopy measurements for symmetric cells featuring cathodes cyclized at different temperatures after resting for a week. The impedance of the P-280-MYS cathode after self-activation was the lowest, indicating that the N-doped carbon surrounding the active materials lowered the charge-transfer resistance (*R*_*ct*_) of the cathode (Fig. 3b and c). Moreover, the N-doped carbon particles captured Cu ions, preventing their migration outside the carbon coating, and acted as active sites for the recrystallization of CuCl_2_ and CuCl during charging-discharging.

### Electrochemical performance

**Fig. 4:**
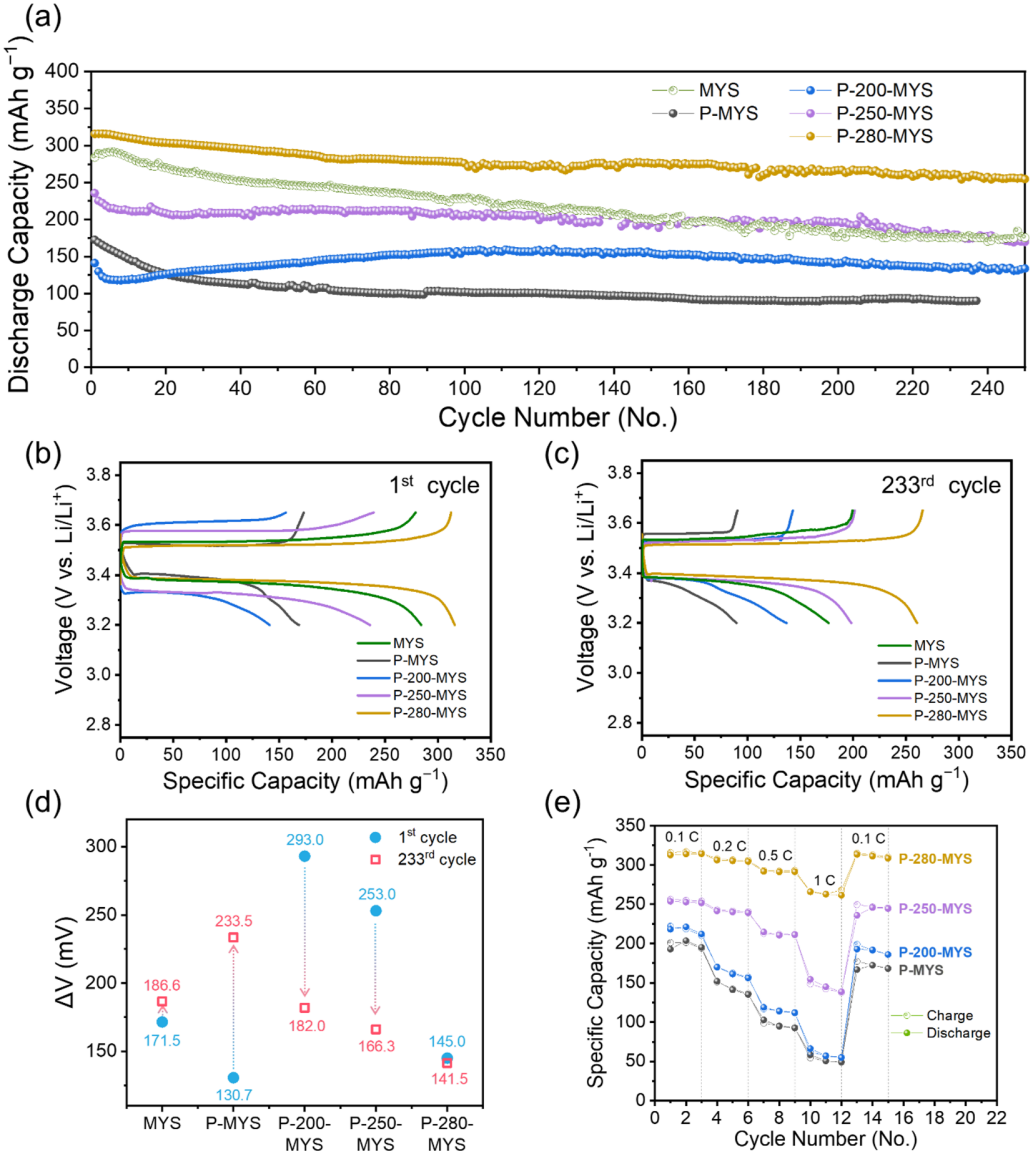
Electrochemical performance of cathodes. **a** Cyclic performance. Galvanostatic voltage profiles at the **b** 1st and **c** 233rd cycles of the multi-yolk–shell (MYS)-CuO cathodes without PAN (MYS), with non-cyclized PAN (P-MYS), and with PAN cyclized at 200, 250, and 280 °C (P-200-MYS, P-250-MYS, and P-280-MYS, respectively) at 0.2 C. **d** Potential differences calculated at a depth of discharge of 50% and state of charge of 50% using the galvanostatic voltage profiles in **b** and **c**. **e** Rate capability of the cathodes at 0.1, 0.2, 0.5, and 1 C (1 C = 300 mA g^− 1^ for CuO).

The electrochemical performances of the MYS, P-MYS, P-200-MYS, P-250-MYS, and P-280-MYS cathodes for Li metal dual-ion batteries with SO_2_-in-salt electrolytes are presented in Fig. 4. The discharge capacity and capacity retention of the batteries significantly depended on the cathode resistivity and *R*_*ct*_. The initial discharge specific capacity of the P-280-MYS cathode was the highest (315.9 mAh g^-1^) among all analyzed cathodes. This value was comparable to the theoretical capacity of 336.9 mAh g^-1^ at 0.2 C, which corresponded to an energy density of ~ 1295 Wh kg_CuO_^-1^. Moreover, the P-280-MYS cathode exhibited excellent cycling stability, with a capacity retention of ~ 84% (~ 266.8 mAh g^-1^) after 200 cycles. The discharge specific capacity of the MYS cathode faded faster than that of the P-280-MYS cathode, dropping from 284.2 mAh g^-1^ in the 1st cycle to 177.0 mAh g^-1^ in the 200th cycle, with a capacity retention rate of only 62.3%. The P-200-MYS and P-250-MYS cathodes exhibited good capacity retention in the 200th cycle, compared to their highest capacities of 87.9% and 83.6%, respectively (the highest specific capacity was 160.2 mAh g^-1^ in the 124th cycle for P-200-MYS and 235.7 mAh g^-1^ in the first cycle for P-250-MYS). We speculated that the O-containing groups formed by heat treatment in an oxygenated environment (found in the FT-IR analysis, as shown in Fig. 2d) may be responsible for the improved cycle retention of P-200-MYS, P-250-MYS, and P-280-MYS cathodes, as PAN functionalized with negatively charged groups was reported to be an adsorbent for metal ions that can suppress the separation of Cu ions from the cathode [35–37]. Meanwhile, the O-containing groups and residual C ≡ N groups of P-200-MYS and P-250-MYS limited their electrical conductivity, which contributed to the large initial Δ*V* values of the cathodes, as illustrated in their voltage profiles (Fig. 4b). This effect was overcome as the cycles progressed (Fig. 4c). The Δ*V* values were calculated at a depth of discharge (DOD) of 50% and a state of charge (SOC) of 50%. In addition, C ≡ N groups with delocalized electron structures, typically repel anions and attract cations, thereby hindering ion transport during charging-discharging. This could be the reason why the Δ*V* values of P-MYS increased after cycling. (Figs. 2a and 4c)

The charge-discharge profiles of the MYS-CuO cathode during the 1st and 233rd cycles are presented in Fig. 4b and c, respectively. The cell voltage during discharging (*V*_*dis*_) and charging (*V*_*ch*_) is expressed as follows:

6 $${V}_{dis}={V}_{emf}-Ir$$

and

7 $${V}_{ch}={V}_{emf}+Ir$$

where *V*_*emf*_ is the electromotive force of the battery, *I* is the current, and *r* is the internal resistance of the cell, which includes contact resistance, electrolyte resistance, activation polarization, and concentration polarization of the cathode.

The fluctuations in the Δ*V* value suggested that the *r* value changed during cycling. During heat treatment, the C ≡ N groups of PAN were converted into amide groups owing to cyclization or the so-called “stabilization” process. Cyclized PAN is an N-doped carbon material, and its electrical conductivity is higher than that of the raw PAN (P-MYS). Furthermore, the N-doped carbon with delocalized sp^2^ π-bonds during cyclization improves the adhesion to the active material [38, 39], leading to faster ion transfer and compatible mechanical resiliency. Because all the C ≡ N groups of PAN heat-treated at 280 °C were converted into amide groups, P-MYS-280 was the only cathode that retained a high discharge capacity over 200 cycles, and its Δ*V* value was constant, as presented in Fig. 4d.

The rate capabilities of the cathodes are shown in Fig. 4e. The charge and discharge rate capabilities were measured at several current rates between 0.1 and 1 C and in the voltage range of 3.2–3.7 V vs. Li^+^/Li. The charge and discharge rates were maintained constant. The capacity of P-280-MYS was the highest at all C-rates and remained above 250 mAh g^-1^ even at a high rate of 1 C, whereas the other cathodes exhibited relatively poor rate properties.

**Fig. 5:**
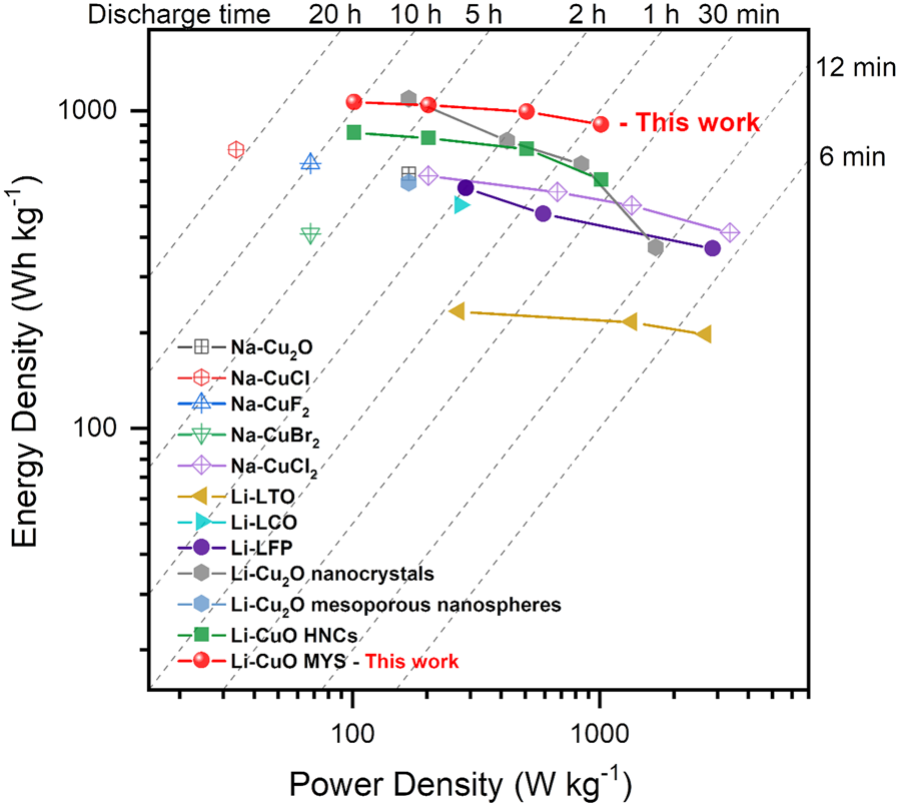
Ragone plot for comparison of the energy density and power density among state-of-the-art cathodes of Li/Na-SO_2_ battery with optimized rate performance as reported in the literatures.[6, 7, 9–11, 40, 41]

In the Ragone plot shown in Fig. 5, the rate performance of the MYS-CuO cathode (P-MYS-280) is compared with that of state-of-the-art cathode materials for Li/Na-SO_2_ batteries. The simple nanosizing improved the energy density as a result of the Li-Cu_2_O nanocrystals [9] and Li-CuO hollow nanocubes (HNCs) [11] shown in Fig. 5; however, as discussed earlier, the improvements in the power density are limited. The energy density of MYS-CuO, which is a nanocluster, was over 900 Wh kg^-1^ at any given power density. Moreover, the C-rate was maintained at 1 C during charging, and there was no significant deterioration in the discharge capacity (the energy density and power density at 1 C were 903 Wh kg^-1^ and 1020 W kg^-1^, respectively).

### Morphology evolution of PAN-coated MYS-CuO during charge-discharge

**Fig. 6:**
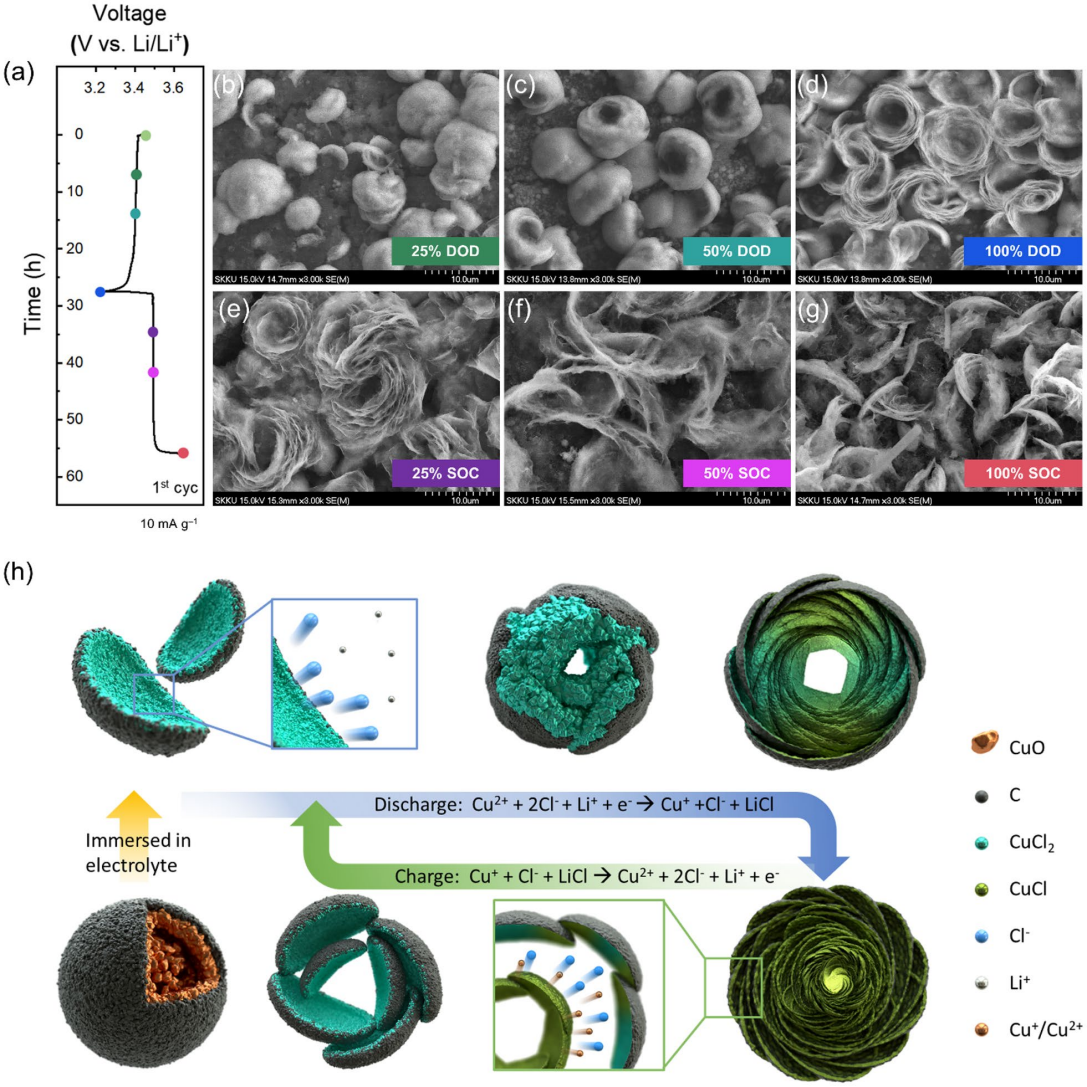
Evolution of particle morphology of the PAN-coated MYS-CuO cyclized at 280 °C (P-280-MYS) used as the cathode during charging-discharging. **a** Voltage profile of the P-280-MYS cathode at a current density of 10 mA g^− 1^ during the first charge-discharge cycle. Surface morphology of the P-280-MYS cathode at depths of discharge (DODs) of **b** 25%, **c** 50%, and **d** 100% and states of charge (SOCs) of **e** 25%, **f** 50%, and **g** 100%. **h** Schematic of the overall morphological evolution of the active material of the P-280-MYS cathode during the first charge-discharge cycle

Changes in the microstructure of the P-280-MYS cathode during charging and discharging were evaluated using FE-SEM to elucidate the reasons for its excellent electrochemical performance. The P-280-MYS cathodes were disassembled at DODs and SOCs of 25%, 50%, and 100% of the first cycle (Fig. 6a), and their corresponding FE-SEM images are presented in Fig. 6b–g and Additional file 1: Fig. S7a. During the early discharge stage (DOD of 25%), the buds that sprouted from the seeds (MYS) were planted in the field (cathode) and convexly protruded on the cathode surface. Thereafter, the buds gradually swelled and opened as the DOD increased to 50% and turned into blooming flowers when the cathode was completely discharged at a DOD of 100% (Fig. 6b–d and Additional file 1: Fig. S7a). The XRD patterns and energy-dispersive X-ray spectroscopy mappings of the cathodes revealed that the petals produced during discharging consisted of CuCl and LiCl (Additional file 1: Fig. S8). During charging, the CuCl and LiCl petals at the center of the flower gradually disappeared (Fig. 6e and f). Finally, the active material returned to its initial shape at 100% SOC (Fig. 6g and Additional file 1: Fig. S7a). The overall morphology evolution of the active material particles during the first charge–discharge cycle is illustrated in Fig. 6h. The MYS-CuO microspheres surrounded by cyclized PAN were converted to CuCl_2_ through a self-activation process. At this point, recrystallization caused a significant change in the volume of the initial yolk–shell microspheres, which split and resemble germinating seeds. Upon discharge, the Cl^–^ ions originating from CuCl_2_ combined with the Li^+^ ions in the electrolyte to form LiCl, and the reduced monovalent Cu^+^ ions recrystallized with the residual Cl^–^ ions to form CuCl. The initial CuCl_2_ particles presented curved shapes, with the convex part covered by the cyclized PAN and the concave part in direct contact with the electrolyte. As a result, Cl^–^ ions easily diffused into the concave parts of the CuCl_2_ particles, and the byproducts generated via discharge grew inward and exhibited a bud-like morphology. When the generated discharge products grew larger than the space inside the buds, the discharge product particles bloomed outward to form a flower-like morphology. During charging, the byproducts disappeared from the center of the flower shape and returned to the sprout shape, similar to the process described above. It is possible to provide a variable space inside the concave area of the active material and ensure constant conductive pathways through the convex side.

## Conclusion

The separation of active cathode materials during the ion-exchange and recrystallization reactions of the self-activation and cyclic conversion steps limited the specific capacity and decreased the cycle retention. We demonstrated that the 1 μm MYS-CuO microspheres served as an excellent active cathode material. Such morphological design ensured a fast CuO self-activation reaction to CuCl_2_ and prevented the conductivity of the KB conductive material from decreasing owing to the dispersed and rapid reactions. Oxidative cyclized PAN containing N-doped delocalized sp^2^ π-bonds was used as the recrystallization catalyst/template for the cathode because of the effect of conductive carbon on the self-activation and cyclic conversion of the active cathode materials. The P-280-MYS cathode exhibited an initial specific capacity of 315.9 mAh g^− 1^ (93.8% of the theoretical value) at 0.2 C, an energy density of 1295.19 Wh kg^− 1^, and a retention rate of 84.46% after 200 cycles. The flower-blooming-like cathode morphology observed during the discharging and charging of the P-280-MYS cathode demonstrated that the cyclized PAN served as a template for recrystallization of the active material, minimizing its separation and improving the electrochemical performance of the cathode. Our results promote the use of non-flammable Li metal dual-ion battery systems with excellent specific capacities and cycle retention performances. Moreover, our study provides constructive perspectives for the optimization of conversion cathodes through reactions that involve significant morphological changes.

## Supplementary Information

**Additional file 1: Figure S1.** Schematic of conversion reaction for the cathode active materialsduring resting and cycling. Crystal structure and parameters of CuO, CuCl, andCuCl_2_ are illustrated. **Figure S2.** FE-SEM images of as-prepared (a) MYS-CuO and (b) CuO hollow nanocubes(HNCs). (c-h) FE-SEM images for electrodes after immersion in electrolyte,including no carbon electrodes with (c) MYS-CuO and (d) CuO HNCs, KB containingelectrodes (CuO : KB : PTFE = 6 : 2 : 2) with (e) MYS-CuO (MYS-CuO+KB) and (f)CuO HNCs (CuO HNCs+KB), and cyclized PAN containing electrodes (CuO : KB : PAN: PTFE = 6 : 2 : 1 : 1) with (g) MYS-CuO (MYS-CuO+KB+PAN) and (h) CuO HNCs (CuOHNCs+KB+PAN). **Figure S3.** XRD patterns of as-prepared MYS-CuO, and electrodes with differentcompositions after immersion in electrolyte. As shown in Figure. S2c-fand Figure. S3, after immersion in the electrolyte, more, thicker and longerCuCl_2_ nanorods or nanowires were observed on CuO cathodes containing0-D KB compared to carbon-free CuO cathodes, indicating that KB promoted theconversion of CuO to CuCl_2_. In the absence of KB, well-dispersednano-sized CuO HNCs were converted to needle-like CuCl_2_ more rapidlycompared to 1-μm MYS-CuO. **Figure S4.** (a-b) SEM images for the broken-particles of asprepared MYS-CuO. (c-d) Low magnification SEM images of as prepared MYS-CuO. **Figure S5.** N 1s XPS profiles for MYS-CuO electrode with PAN cyclized at differenttemperatures. **Figure S6.** Differential scanning calorimetry thermograms ofPAN powder, PAN-coated MYS-CuO, and bare MYS-CuO (a) before and (b) afterheat treatment at 280 ℃ from 25 to 500℃ with air flowing at a heating rate of 2℃ min^-1 ^. **Figure S7.** (a)FE-SEM images of bare P-280-MYS electrode and P-280-MYS electrodes afterresting and 1st discharge and charge. (b) FE-SEM images of P-280-MYSelectrode after 50th discharge. **Figure S8.** (a) XRD patterns for raw, rested, discharged, and charged P-280-MYS electrodes. FE-SEM and EDS mapping imagesof (b) rested, (c) fully discharged, and (d) fully charged P-280-MYS electrodes.

## Data Availability

The datasets used and/or analyzed during the current study are available from the corresponding author on reasonable request.
