# Supplementary material for: Reversible flowering of CuO nanoclusters via conversion reaction for dual-ion Li metal batteries
Source: Nano Converg. 2023 Jan 13;10:4. doi: 10.1186/s40580-022-00353-3 (PMC9839906; doi:10.1186/s40580-022-00353-3)
Supplement: Supplementary file 1 — Additional file 1: Figure S1. Schematic of conversion reaction for the cathode active materialsduring resting and cycling. Crystal structure and parameters of CuO, CuCl, andCuCl2 are illustrated. Figure S2. FE-SEM images of as-prepared (a) MYS-CuO and (b) CuO hollow nanocubes(HNCs). (c-h) FE-SEM images for electrodes after immersion in electrolyte,including no carbon electrodes with (c) MYS-CuO and (d) CuO HNCs, KB containingelectrodes (CuO : KB : PTFE = 6 : 2 : 2) with (e) MYS-CuO (MYS-CuO+KB) and (f)CuO HNCs (CuO HNCs+KB), and cyclized PAN containing electrodes (CuO : KB : PAN: PTFE = 6 : 2 : 1 : 1) with (g) MYS-CuO (MYS-CuO+KB+PAN) and (h) CuO HNCs (CuOHNCs+KB+PAN). Figure S3. XRD patterns of as-prepared MYS-CuO, and electrodes with differentcompositions after immersion in electrolyte. As shown in Figure. S2c-fand Figure. S3, after immersion in the electrolyte, more, thicker and longerCuCl2 nanorods or nanowires were observed on CuO cathodes containing0-D KB compared to carbon-free CuO cathodes, indicating that KB promoted theconversion of CuO to CuCl2. In the absence of KB, well-dispersednano-sized CuO HNCs were converted to needle-like CuCl2 more rapidlycompared to 1-μm MYS-CuO. Figure S4. (a-b) SEM images for the broken-particles of asprepared MYS-CuO. (c-d) Low magnification SEM images of as prepared MYS-CuO. Figure S5. N 1s XPS profiles for MYS-CuO electrode with PAN cyclized at differenttemperatures. Figure S6. Differential scanning calorimetry thermograms ofPAN powder, PAN-coated MYS-CuO, and bare MYS-CuO (a) before and (b) afterheat treatment at 280 ℃ from 25 to 500℃ with air flowing at a heating rate of 2℃ min-1 . Figure S7. (a)FE-SEM images of bare P-280-MYS electrode and P-280-MYS electrodes afterresting and 1st discharge and charge. (b) FE-SEM images of P-280-MYSelectrode after 50th discharge. Figure S8. (a) XRD patterns for raw, rested, discharged, and charged P-280-MYS electrodes. FE-SEM and EDS mapping imagesof (b) rested, (c) fully discharged, and [file 40580_2022_353_MOESM1_ESM.docx]

Reversible flowering of CuO nanoclusters via conversion reaction for dual-ion Li metal batteries

Siying Li^1,2^, Jung-Hun Lee^2^, Soo Min Hwang^2, *^, and Young-Jun Kim ^2,3 *^

^1^ School of Mechanical and Automotive Engineering, Guangxi University of Science and Technology, Liuzhou 545616, China

^2^ SKKU Advanced Institute of Nanotechnology (SAINT), Sungkyunkwan University, Suwon 16419, Republic of Korea

^3^ SKKU Institute of Energy Science and Technology (SIEST), Sungkyunkwan University, Suwon 16419, Republic of Korea

**Natural activation reactions**

Main reaction:

$3CuO+2LiAlCl_{4}\to3CuCl_{2}+2LiCl+Al_{2}O_{3}$

$\Delta G^{^{\circ}}=-342.48 kJ mol^{-1}$ (S1)

Side reactions:

$$CuO+LiAlCl_{4}+\frac{1}{2}SO_{2} \to CuCl+\frac{1}{2}Li_{2}SO_{4}+AlCl_{3}$$

${\Delta G}^{^{\circ}}=-61.22 kJ mol^{-1}$ (S2)

$3Li_{2}SO_{4}+2AlCl_{3}\to6LiCl+{Al}_{2}(SO_{4})_{3}$

$\Delta G^{^{\circ}}=-562.2 kJ mol^{-1}$ (S3)

$3CuO+2AlCl_{3}\to3CuCl_{2}+Al_{2}O_{3}$.

$\Delta G^{^{\circ}}=-570.86 kJ mol^{-1}$ (S4)

$$CuCl_{2}+\frac{1}{3}Al_{2}O_{3}+\frac{1}{2}SO_{2} \to CuCl+\frac{1}{3}AlCl_{3}+{\frac{1}{6}Al}_{2}(SO_{4})_{3}$$

$\Delta G^{^{\circ}}=-60.8 kJ mol^{-1}$ (S5)


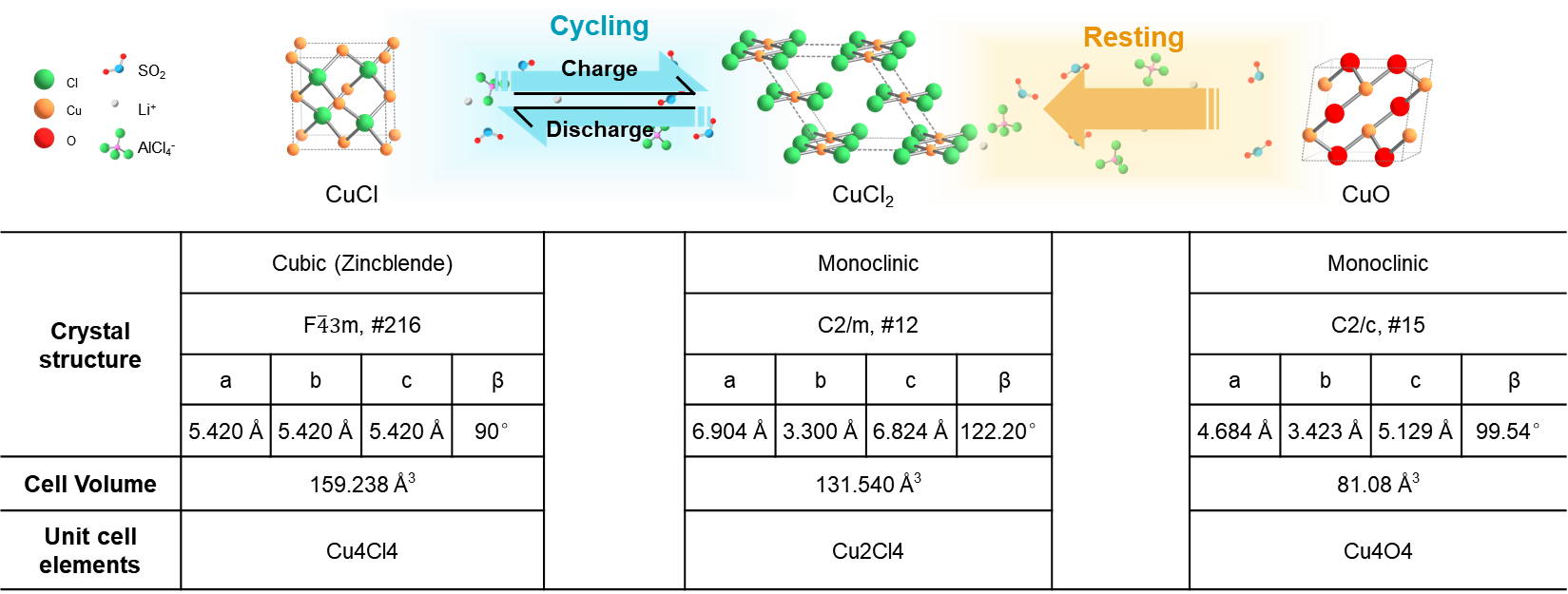


**Figure S1** Schematic of conversion reaction for the cathode active materials during resting and cycling. Crystal structure and parameters of CuO, CuCl, and CuCl_2_ are illustrated.


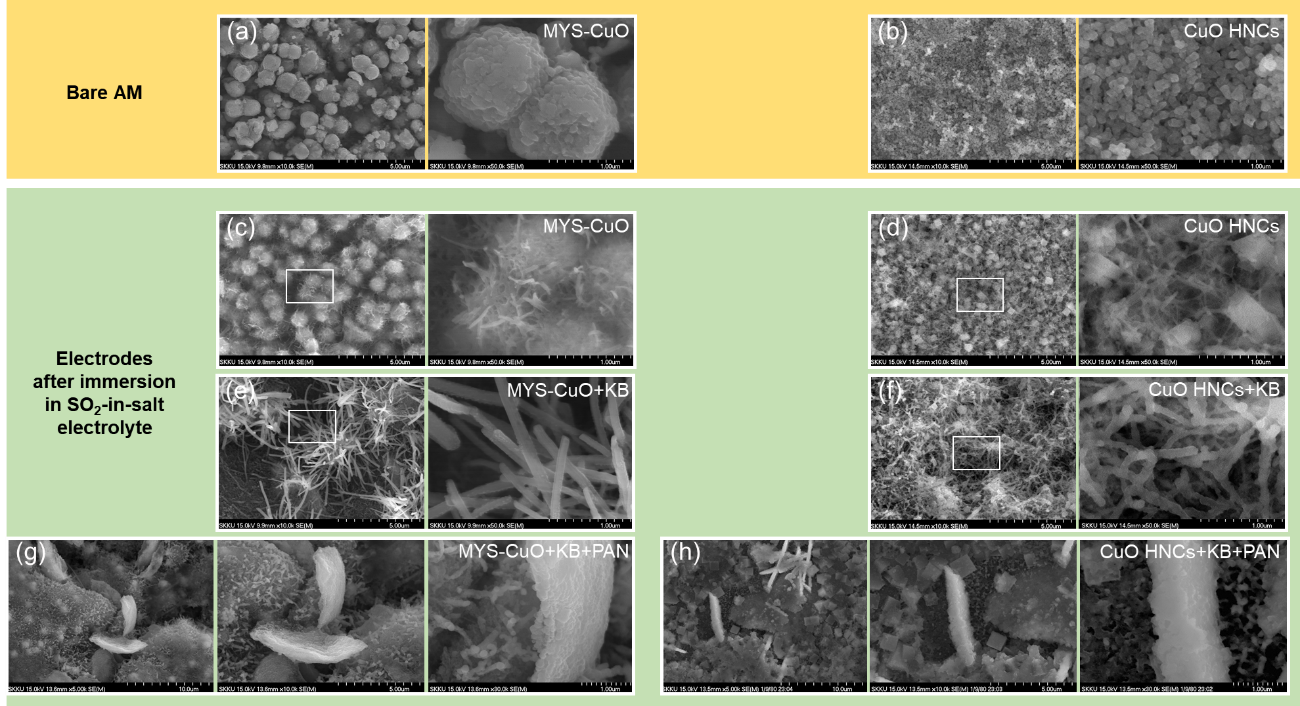


**Figure S2** FE-SEM images of as-prepared (a) MYS-CuO and (b) CuO hollow nanocubes (HNCs). (c-h) FE-SEM images for electrodes after immersion in electrolyte, including no carbon electrodes with (c) MYS-CuO and (d) CuO HNCs, KB containing electrodes (CuO : KB : PTFE = 6 : 2 : 2) with (e) MYS-CuO (MYS-CuO+KB) and (f) CuO HNCs (CuO HNCs+KB), and cyclized PAN containing electrodes (CuO : KB : PAN : PTFE = 6 : 2 : 1 : 1) with (g) MYS-CuO (MYS-CuO+KB+PAN) and (h) CuO HNCs (CuO HNCs+KB+PAN)


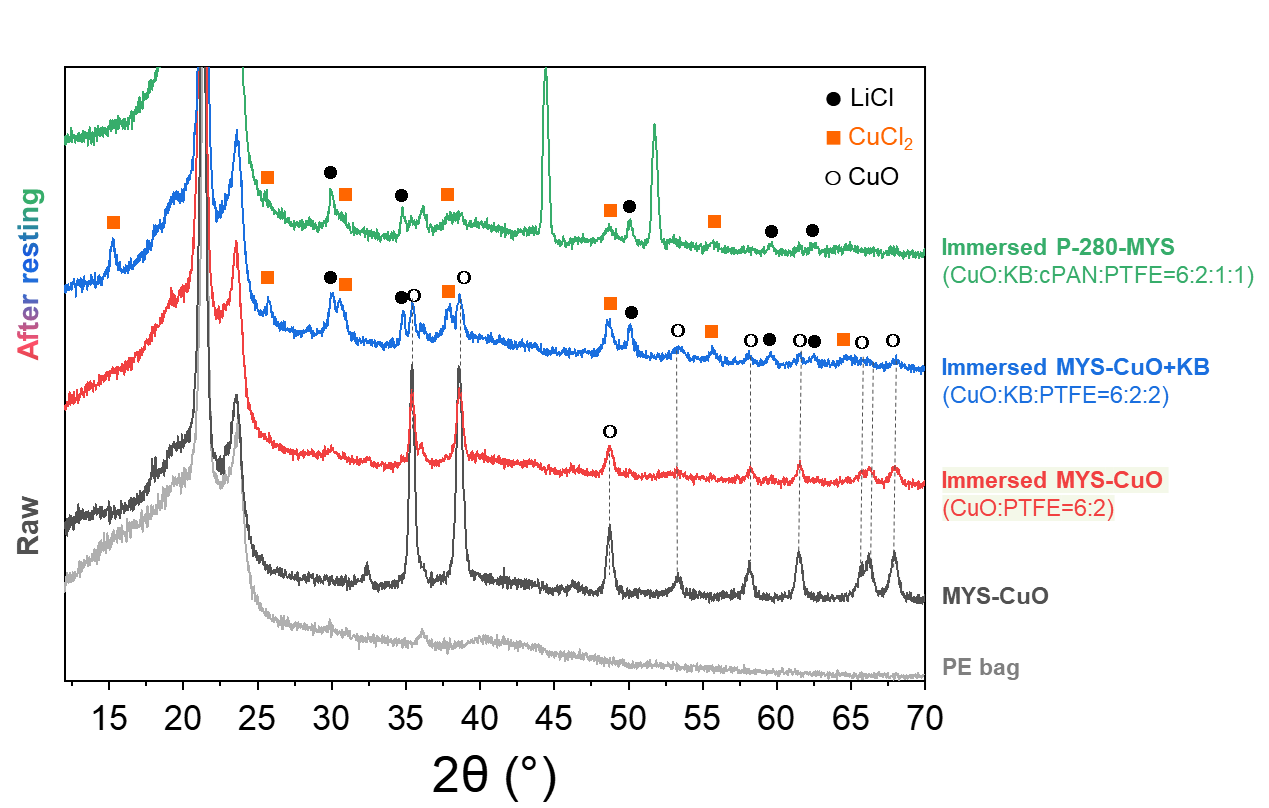


**Figure S3** XRD patterns of as-prepared MYS-CuO, and electrodes with different compositions after immersion in electrolyte.

As shown in Figure. S2c-f and Figure. S3, after immersion in the electrolyte, more, thicker and longer CuCl_2_ nanorods or nanowires were observed on CuO cathodes containing 0-D KB compared to carbon-free CuO cathodes, indicating that KB promoted the conversion of CuO to CuCl_2_. In the absence of KB, well-dispersed nano-sized CuO HNCs were converted to needle-like CuCl_2_ more rapidly compared to 1-μm MYS-CuO.


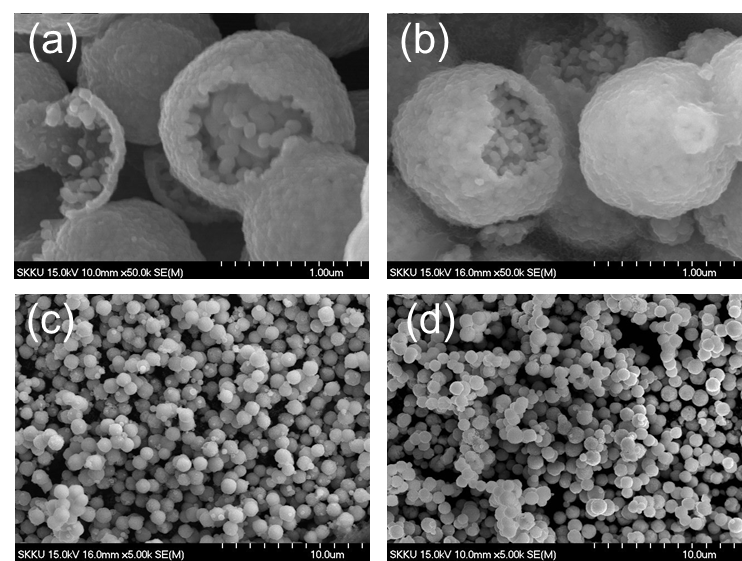


**Figure S4.** (a-b) SEM images for the broken-particles of as prepared MYS-CuO. (c-d) Low magnification SEM images of as prepared MYS-CuO.


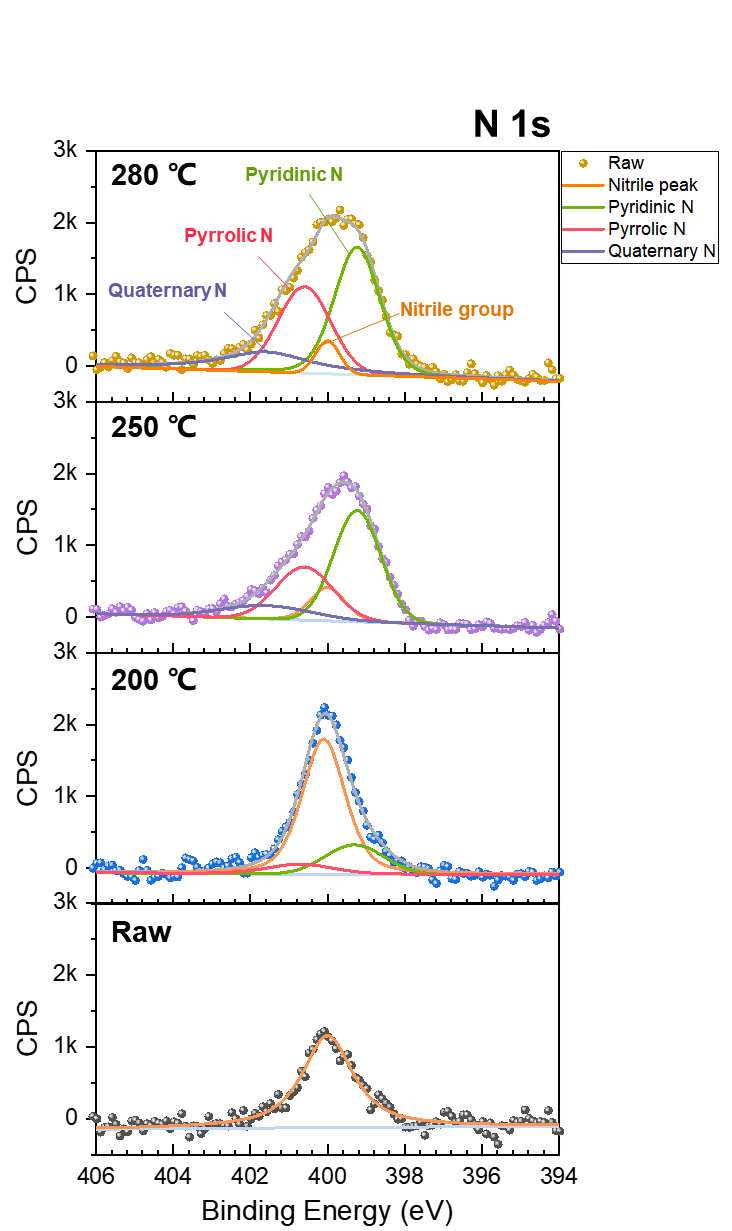


**Figure S5** N 1s XPS profiles for MYS-CuO electrode with PAN cyclized at different temperatures.


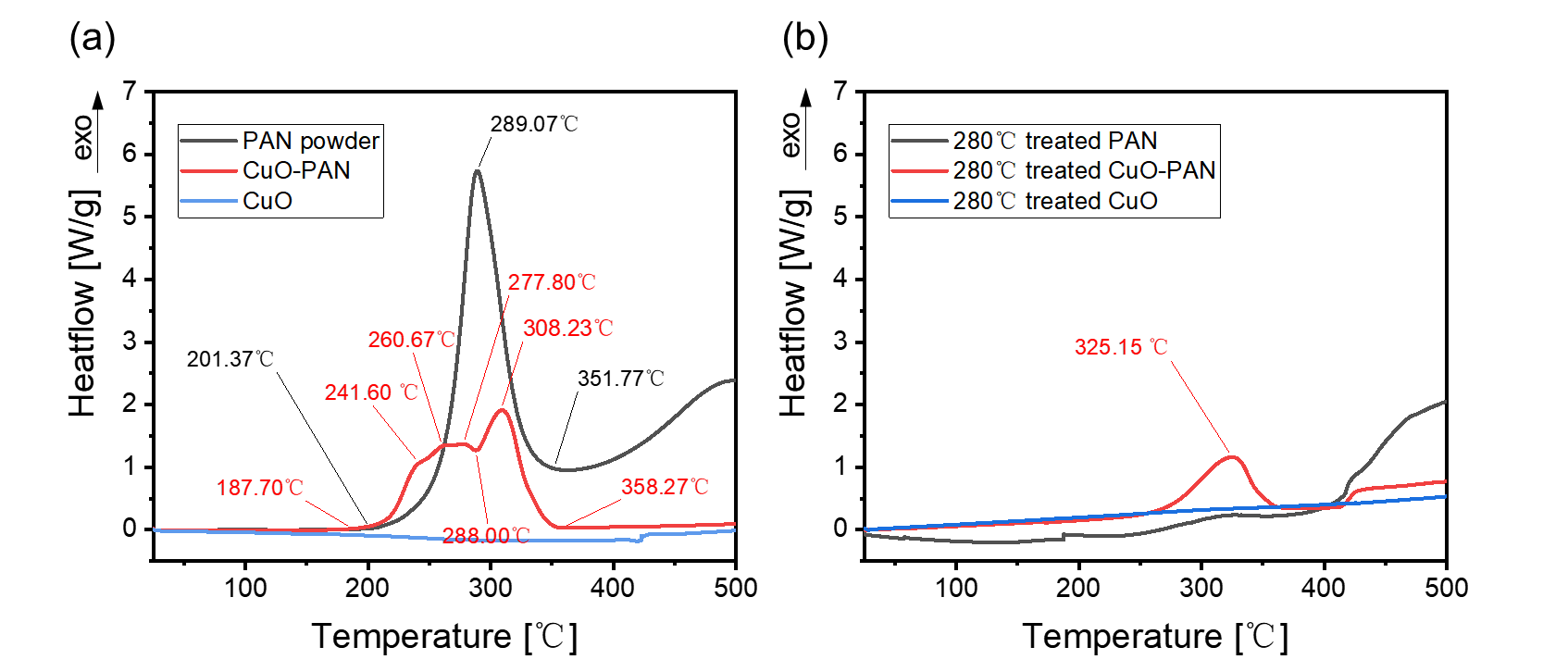


**Figure S6** Differential scanning calorimetry thermograms of PAN powder, PAN-coated MYS-CuO, and bare MYS-CuO (a) before and (b) after heat treatment at 280 ℃ from 25 to 500℃ with air flowing at a heating rate of 2 ℃ min^-1^。


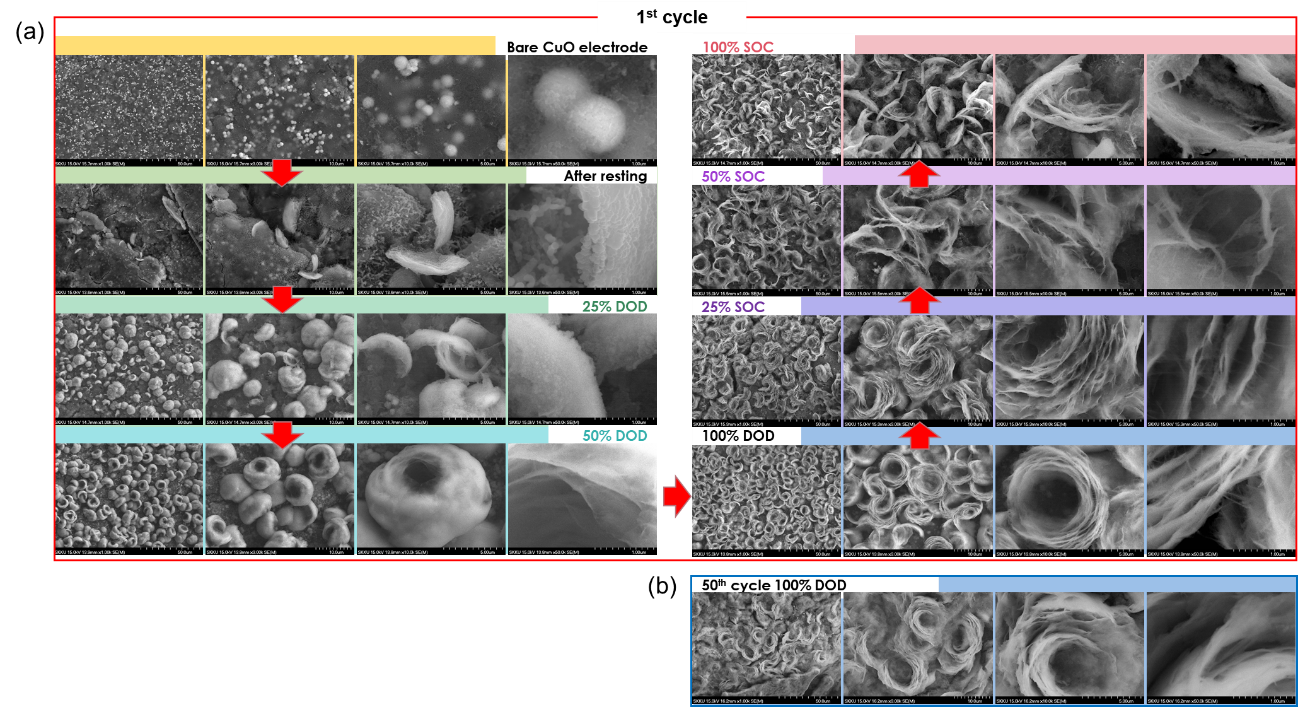


**Figure S7** (a) FE-SEM images of bare P-280-MYS electrode and P-280-MYS electrodes after resting and 1^st^ discharge and charge. (b) FE-SEM images of P-280-MYS electrode after 50^th^ discharge.


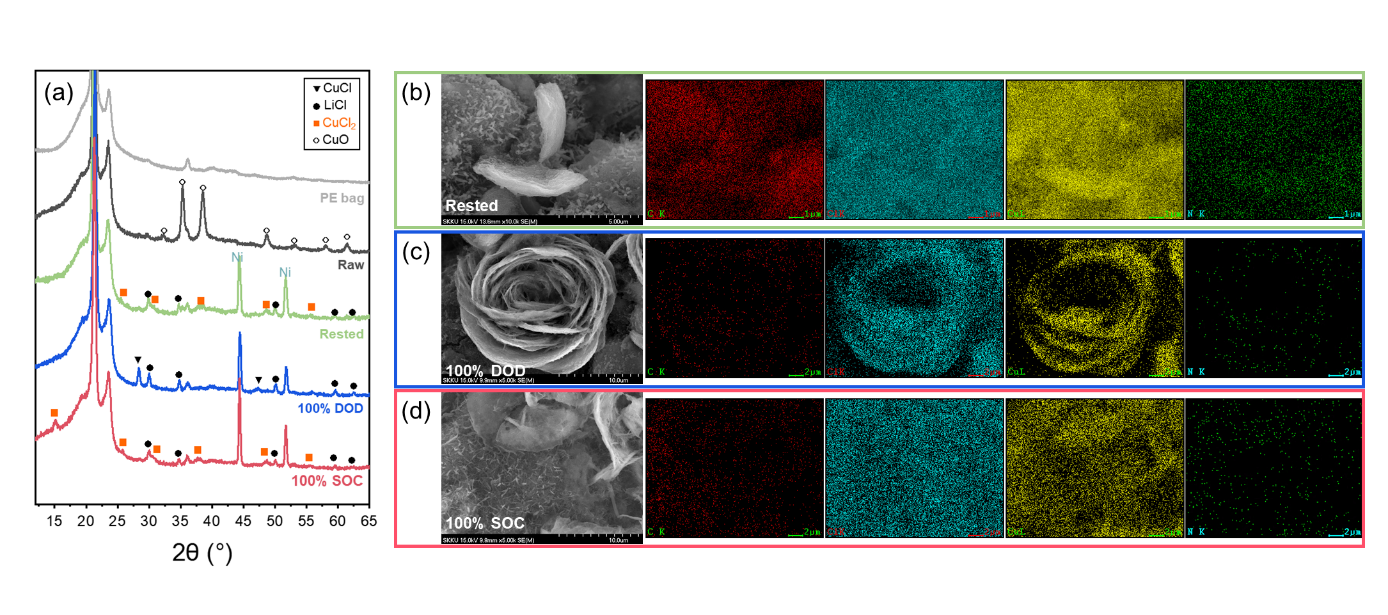


**Figure S8** (a) XRD patterns for raw, rested, discharged, and charged P-280-MYS electrodes. FE-SEM and EDS mapping images of (b) rested, (c) fully discharged, and (d) fully charged P-280-MYS electrodes.
